# HTA Agencies Need Evidence-Informed Deliberative Processes Comment on "Use of Evidence-Informed Deliberative Processes by Health Technology Assessment Agencies Around the Globe"

**DOI:** 10.34172/ijhpm.2020.22

**Published:** 2020-02-19

**Authors:** Michael Schlander

**Affiliations:** ^1^German Cancer Research Center (Deutsches Krebsforschungszentrum, DKFZ), & University of Heidelberg, Heidelberg, Germany.; ^2^Institute for Innovation & Valuation in Health Care (InnoValHC), Wiesbaden, Germany.

**Keywords:** Accountability for Reasonableness, Legitimacy, Cost-Effectiveness, Guidance, Social Preferences, Decision-Making

## Abstract

There are at least two reasons why health technology assessment (HTA) agencies need to seek process-based solutions to support the legitimacy of healthcare resource allocation, ie, (i) in pluralistic societies, the existence of often conflicting and incommensurable claims (ie, the "fragmentation of value") and the lack of a broadly accepted, ethically defensible analytical framework, and (ii) the well-documented loopholes of the conventional logic of cost-effectiveness (CE) with its reductionist concept of allocative efficiency, which fails to reflect the distributive dimension of resource allocation decisions in collectively financed health schemes.

## Background

In a recent issue of *IJHPM*, Wija Oortwijn, Maarten Jansen, and Rob Baltussen (OJB)^1^ reported insights from a survey that they conducted among international health technology assessment (HTA) agencies, which had been designed to explore their use of “evidence-informed deliberative processes” (EDPs). EDPs have been proposed “to improve guidance to HTA organizations to improve their processes towards more legitimate decision-making.”^1^ As such, they are closely related to schools of thought in political theory that emphasize the role of fair and reasonable deliberative processes as a precondition for the legitimacy of political decisions in a democratic society. Following contemporary philosophers, prominently including John Rawls^2^ and Jürgen Habermas,^3,4^ the use of reason implies the collection of factual information, competing arguments, and different viewpoints.

Legitimate decisions in a pluralistic society then become the result of agreement based on the balancing of the value judgments of fair-minded people with diverse perspectives, a balancing that occurs during the process of deliberation prior to actual decision-making. It is broadly held by deliberativists that maximum inclusion of citizens and perspectives should result in maximum legitimacy and reasonableness of the outcomes – an assumption that may conflict with the need to establish rules of argumentation and the capacity of (at least some) citizens to be reasonable and cooperative. Then, according to Habermas and other deliberativist scholars, in socially integrated contexts an ideal process can be expected to “generate wide, though of course not complete, actual consensus on political outcomes.”^5^

Note that deliberative or discursive processes as a source of legitimate decisions are fundamentally different from the simple aggregation of (selfish) preferences, for example by relying on individual maximum willingness-to-pay as a measure of their strength^6^ as in cost benefit analyses grounded in economic welfare theory.^7^ In the case of cost-effectiveness (CE) or “cost-utility” evaluation, which has become a central component of many HTA processes, health gains are aggregated by additive summing-up, with length of life and preference-weighted quality of life as the principal sources of value considered for analysis. Both dimensions are then integrated in one simple metric, usually the quality-adjusted life year (QALY). Assuming a (hypothetical) willingness-to-pay (or a shadow price, if a supply-side perspective is preferred) for a QALY leads to a CE threshold value, which translates into simple decision rules and analytical convenience.

Over the last five decades, a substantive literature has evolved describing a set of broadly accepted conventions and prescribing how to apply the logic of CE as a tool to inform healthcare resource allocation decisions.^7^ In essence, they are based on a ranking of condition/intervention pairs on grounds of their respective incremental cost-effectiveness ratios (ICERs), implying increasing efficiency and hence social desirability of a medical technology with decreasing ICERs. The underlying premise here is “that it is ethical to be efficient, since to be inefficient implies failure to achieve the ethical objective of maximising health benefits from available resources.”^8^

## Limitations of the Conventional Logic of Cost-Effectiveness

The central role of ICERs as a yardstick of “efficiency” has been seriously challenged by economists, not least for the ratio falling short of providing policy-makers with any information about the size of its numerator and denominator. By implication, the ICER cannot provide any useful information about the opportunity cost of adopting a new healthcare program from the perspective of a policy-maker acting on behalf of the members of a collectively financed health scheme.

For example, Canadian health economists Stephen Birch and Amiram Gafni have repeatedly drawn attention to the arbitrary nature of threshold ICERs.^9,10^ They pointed out that adopting a CE decision rule (implying acceptance of new technologies with ICERs that are deemed acceptable in a given context and that are not negative) may turn out to be “a prescription for uncontrolled growth in expenditures.”^9^ By implication, under a strict budget constraint some already funded technologies would have to be defunded. In an ideal world according to the CE paradigm, the displaced technologies indeed should be the least cost-effective ones – but how do we know this happens in reality?^10-12^

While the displacement concern might be more of a “technical nature,” the linearity assumption inherent in the ICER construct is much more contentious. It implies that the social value of adding a new medical intervention is being assumed to be strictly proportional to the number of persons benefiting, similar to applying a standard act utilitarian calculus. The consequences are far reaching, as adoption of the underlying logic as a basis for decision-making – informed by presumably value-free evidence – would necessarily lead to the disen­franchisement of patients with rare disorders from any chance to get access to new effective treatment options.^13^ One of the major reasons frequently cited for this is the high fixed/low variable cost structure of the research-based biopharmaceutical industry and the need of manufacturers to recoup fixed expenditures from small patient numbers.^13^

The consequence of systematically leaving behind groups of patients with rare and ultra-rare disorders would form a stark contrast to the increasingly well-documented wish of citizens to share resources in a way which does not exclude patients who require services that are not “efficient” (ie, cost-effective), such as many orphan medicinal products and treatments for patients with ultra-rare diseases.^14^ Vice versa, experience shows that some services are not covered by national health schemes despite having been shown to be cost-effective,^15^ most plausibly (at least in part) because of a public preference for giving a higher priority to interventions for patients in more severe initial health states, ie, with a higher need for effective care.^16^ Apparently some outcomes of the logic of CE violate prevailing moral norms and intuitions, failing to pass tests of reflective equilibrium.^13^

Abstracting from a range of further restrictive assumptions and conventions inherent in the conventional logic of CE, potential explanations for the violation include the notions (*i*) that there is a perfect correspondence between selfish *ex ante* preferences and social value, (*ii*) that all relevant components of costs and value are comprehensively captured in the QALY model, (*iii*) that the dominant social objective of a health scheme is to maximize the sum-total of health (or QALYs) produced, and (*iv*) that the QALY itself is an economic measure of health-related utility – despite effectively imposing a linear utility function instead of diminishing marginal utility over time.^10,17,18^ This last assumption is evident because QALYs are computed by way of simple additive aggregation of utility-adjusted time intervals.^17,18^ The underlying assumptions are either not supported by empirical tests, or have even been shown to be “descriptively flawed”^19^: they must be considered empirically falsified when judged against the growing literature on citizens’ social preferences and value judgments.^14,20^

Against this background it is noteworthy that senior executives of the National Institute for Health and Care Excellence (NICE) in England, which is perceived by many as the prime example for the implementation of the logic of CE, acknowledged that utilitarianism (with of its focus on *aggregate* outcomes) ‘‘has next to nothing to offer in eradicating health inequalities’’^21^ and subscribed explicitly to the principles of accountability for reasonableness (A4R).^21,22^

## The Need for Evidence-Informed Deliberative Processes

So there are at least two reasons for HTA agencies to seek process-based solutions to increase the legitimacy of healthcare resource allocation recommendations, ie,

In pluralistic societies, the reality of often conflicting and incommensurable claims (or the “fragmentation of value,” as described by Thomas Nagel^23^) and the associated lack (or maybe even the impossibility^23,24^) of a broadly accepted, ethically defensible analytical framework, which comprehensively captures both social value and opportunity costs in a collective health scheme in a pluralistic society – a challenge that is aggravated by the normative issues arising from any aggregation mechanism and its inescapable implicit dimension of interpersonal comparison and prioritization.^2-4,23,24^

In particular, the well-documented loopholes of the conventional logic of CE; including its reductionist conceptualization of allocative efficiency in terms of health gain (or QALY) maximization, which fails to reflect the distributional dimension of resource allocation decisions in collectively financed health schemes.^8,10,14,21,24,25^

The search for more and better reasons in a discursive process should lend greater justification and legitimacy to democratic decisions^2-4^; to accomplish this, it must resist the “false dichotomy”^23^ of either adopting unsystematic intuitive judgments or the restrictions of the scope of formal analysis to the conventional CE algorithm and the maximization of a problematic construct of aggregate population health.^24^ In the sphere of HTAs, intended to inform ethically defensible healthcare resource allocation decisions, Norman Daniels and James Sabin called for A4R as one approach to address “unsolved rationing problems,” over which reasonable people continue to disagree despite decades of theoretical debate.^22^ According to Norman Daniels and James Sabin, A4R comprises four conditions, publicity, relevance, appeal, and enforcement.

In this context, the survey by OJB^1^ provides valuable insights into the international level of use of EDPs by HTA agencies. It is reassuring that agencies understand that “EDPs can contribute to the legitimacy of recommendations and/or decisions, eg, by improving the quality, consistency and transparency of the HTA process.”^1^ Since the OJB study was based on self-reports collected from agency staff, their results should however be greeted with a healthy dose of scepticism, as some replies might have been influenced by “social desirability bias,” “self-serving bias,” and possibly even self-praise. Occasionally such phenomena might even extend to commentators, as some of them went as far as to describe NICE as “a form of direct democracy”^26^ and its use of CE as “an exemplar of a deliberative process”^27^ (sic!) – deliberately overlooking studies that found NICE to fall short on all A4R conditions investigated, including the publicity criterion.^28,29^

Shortcomings with regard to publicity ranged from (*i*) the selection of topics for appraisal, withholding of (*ii*) commercial-in-confidence information and of (*iii*) proprietary economic models, as well as (*iv*) restrictive conditions for appeal, to (*v*) distinctly uninformative appraisal committee meeting minutes.^28^ Given the gap between highly codified assessment reports, which at NICE are dominated by conventional CE analyses, and the EDP-type nature of the appraisal process, any limitation of the transparency of the deliberations of the appraisal committees should be a reason for concern.^28,29^ While these remarks are not meant to diminish the accomplishments of NICE, they should serve to inject a dose of caution before taking self-reports of HTA agencies at face value.

## Conclusions and Recommendations

In the absence (and likely impossibility) of a general and complete “grand theory” of healthcare resource allocation, the role of judgment and EDPs in resolving disparate claims and considerations will remain indispensable.^23,24^ While this has been broadly recognized by HTA agencies, their response should not be purely rhetorical. They should actually fulfil the conditions of A4R, in particular with respect to the publicity criterion.

Furthermore, intense efforts should be expected to close the existing gaps between narrowly defined assessment reports and (sometimes much) wider appraisal criteria. Evidence should be presented in a way that facilitates subsequent deliberation by appraisal committees. Arguably, multi-criteria decision analysis frameworks may better support EDPs and stakeholder involvement than currently applied CE evaluations.^30,31^

Health economists, in turn, might wish to spend more effort on closing the gap between societal values and the conventional logic of CE by focusing on the develop­ment and operationalization of evaluation methods that better capture the full range of economic consequences, including relevant social norms and preferences from the perspective of reasonable, well-informed citizens. This might entail consideration of alternative evaluation paradigms.^14,25,32^

## Acknowledgements

The author is grateful for helpful comments on an earlier version of the present commentary by Professor Jeffrey Richardson (Monash University, Melbourne, Australia) and by an anonymous reviewer. The usual disclaimer regarding potential errors applies.

## Ethical issues

Not applicable.

## Competing interests

Author declares that he has no competing interests.

## Author’s contribution

MS is the single author of the paper.
